# The Emerging Perspective of Morphine Tolerance: MicroRNAs

**DOI:** 10.1155/2019/9432965

**Published:** 2019-04-30

**Authors:** Teng J. Zhang, Yong Qiu, Zhen Hua

**Affiliations:** Department of Anesthesiology, Beijing Hospital, National Center of Gerontology, Beijing 100730, China

## Abstract

Morphine has unfavorable side effects including analgesic tolerance. Morphine tolerance counteracts analgesic efficacy and drives dose escalation. The mechanisms underlying morphine tolerance remain disputed, which has prevented the development of therapies to maximize and sustain analgesic efficacy. Morphine tolerance is an adaptive process induced by chronic morphine that has been shown to result from complex alterations at the molecular level with *μ* opioid receptors (MORs), as well as at the synaptic, cellular, and circuit levels. MicroRNAs are noncoding RNAs that have been proposed to regulate gene expression and degradation at the posttranscriptional level, including the MOR, as well as synaptic plasticity and neuroplasticity, in both the peripheral and central nervous systems. This review covers some of the most striking microRNA functions involved in morphine tolerance and presents limitations on our knowledge of their physiological roles.

## 1. Introduction

Opioid analgesics, such as morphine, continue to be the mainstay for managing severe and chronic pain. With the staggering prevalence of pain, the broad use of opioids for pain management has increased markedly over the past decades [1]. However, chronic opioid use can result in analgesic tolerance, hyperalgesia, and other side effects, which seriously affect the safety and comfort of patients [2]. Morphine tolerance is the primary cause of diminished pain control and dose escalation, which makes the related side effects more serious and widespread [1]. Therapeutic strategies that can bolster opioid analgesia while mitigating tolerance are urgently needed to improve patients' safety.

MicroRNAs (miRNAs) are noncoding RNAs of approximately 20 nucleotides in length that block gene expression at the posttranscriptional level by partial complementary binding to the 3′-untranslated region (3′-UTR) of mRNA of target genes in animals, resulting in mRNA degradation or translation inhibition [3]. However, in recent years, there have also been reports of miRNAs activating target mRNAs and upregulating translation, and this process is believed to be related to the cell cycle, i.e., when the cells are in a nonproliferating state, miRNAs may upregulate translation; otherwise, they inhibit translation [4, 5]. However, there are many controversies over this view, and the specific mechanisms remain to be clarified. It is currently believed that miRNAs, as important regulatory factors of epigenetics, may be widely involved in the regulation of various cellular activities including neurobiological responses, such as neuronal growth, metabolism, apoptosis, and synaptic plasticity [6, 7].

Morphine tolerance is an adaptive process that has been proposed to result from complex alterations at the molecular level with *μ* opioid receptors (MORs), as well as at the synaptic, cellular, and circuit levels, in both the peripheral and central nervous systems. Thus, chronic administration of opioids modifies neuronal MOR function through a variety of mechanisms including receptor phosphorylation, signaling, multimerization, and trafficking, which may underlie tolerance to morphine. Morphine administration can lead not only to changes in the expression levels of multiple miRNAs in neuronal tissues or cells but also to differences in the types and degrees of expression of miRNAs induced by different opioids [5, 8–10]. In this review, we highlight and discuss the more recent studies on miRNAs in these adaptive processes.

## 2. Morphine Tolerance Mechanism Overview

Downregulation of MORs and neuroadaptation may be the main mechanisms of morphine tolerance [11, 12]. Downregulation of MORs includes decreased MOR expression and increased degradation, and neuroadaptation includes synaptic plasticity and neuroplasticity [11]. At different transcriptional, posttranscriptional, and epigenetic levels, MOR levels may be regulated, and miRNAs mainly regulate MOR levels at the posttranscriptional level [13]. The miRNAs that may be involved in morphine tolerance are summarized in Table 1.

### 2.1. Morphine Tolerance and MORs

Morphine tolerance refers to the gradual decrease in the potency of a drug following its long-term administration at a fixed dose and usually requires higher and higher doses to maintain the initial level of analgesia [2]. Many types of opioid receptors (e.g., *μ*, *κ*, *δ*) exist in the nervous system, and they are all typical inhibitory G protein-coupled receptors (GPCRs) [30]. MORs are widely distributed in the central (CNS) and peripheral nervous systems, with distribution throughout the CNS including the cerebral cortex, limbic system, thalamus, striatum, hippocampus, locus coeruleus, and superficial laminae of spinal cord dorsal horn [31]. After knocking out the MOR gene, opioids such as morphine not only lose their analgesic effect but no longer cause side effects such as analgesic tolerance, hyperalgesia, and drug addiction [30, 32]. This shows that MORs are crucial for both positive and negative effects of opioids, so the MOR is one of the core components when examining morphine tolerance.

Corder et al. [32] have taken advantage of mouse genetic engineering and successfully generated conditional knockout mice that lack MORs in the peripheral nerve nociceptors but remain intact in the central nervous system. This selective genetic deletion of MORs did not reduce systemic morphine antinociception or result in analgesic tolerance with chronic subcutaneous injection of morphine. Furthermore, co-administration of morphine and methylnaltrexone bromide (MNB; a peripherally restricted MOR antagonist that does not penetrate the blood-brain barrier, i.e., acts only in the periphery) produced the same results as observed in the conditional knockout mice. Notably, microglia that have been shown to be involved in morphine tolerance [33] are still highly activated in conditional knockout mice, suggesting that MORs on peripheral nociceptors are highly likely to be a critical contributor to morphine tolerance [32].

It is generally believed that opioids binding to MORs produce an inhibitory G protein signal that causes analgesia, but side effects including analgesic tolerance, respiratory depression, and constipation may be related to modulation of MOR signaling by *β*-arrestin-2 [30, 34, 35]. The sustained action of opioids results in the upregulation of G protein-coupled receptor kinase activity, phosphorylation of MORs, and increased affinity for beta-arrestin-2; subsequent recruitment of beta-arrestin-2 results in uncoupling of the G protein from the receptor (i.e., desensitization) followed by internalization of the receptor [12, 36, 37]. Internalization is thought to be a physiologically protective mechanism for the body to avoid long-term sustained activation of MORs; at some point, the receptor can be recycled back to the cell membrane to continue to function, that is, resensitization occurs [12, 37]. However, the fate of receptors after internalization is more complex because MORs may also be transported to the lysosome to be directly degraded, which would result in fewer available receptors at the cell membrane and could intuitively explain cellular tolerance to opioids [11, 36, 37].

### 2.2. Morphine Tolerance and Neuroadaptation

Synaptic plasticity is also well known to participate in the functional regulation of nociceptive pathways [38]. Long-term potentiation (LTP) is the main manifestation of long-term synaptic plasticity. It is a long-lasting increase in signal transmission between two neurons following high-frequency stimulation of a chemical synapse [39]. Electrophysiological studies have demonstrated that opioids not only depress neurotransmission between nociceptors and dorsal horn neurons but can also generate maladaptive plasticity, such as LTP, which may contribute to tolerance [40]. Furthermore, LTP occurs throughout the central nervous system, e.g., in the cerebral cortex, striatum, hippocampus, and amygdala, and LTP is involved in numerous processes including learning, memory, hyperalgesia, drug addiction, and tolerance [38, 41–43]. The presynaptic versus postsynaptic origin of opioid-induced LTP remains disputed, and whether LTP is initiated by MORs activation in nociceptors or spinal neurons is not known [40, 44]. Notably, a recent study suggested that prolonged activation of presynaptic MORs on nociceptors had predominantly pronociceptive effects during chronic morphine exposure and initiated downstream plasticity throughout nociceptive circuits in the central nervous system to drive the development of tolerance [32].

Activated microglia and astrocytes are considered important contributors to morphine tolerance, and inhibition of glial activation can reduce tolerance [33, 45–47]. In terms of mechanisms, many possible pathways describing direct and indirect actions of morphine that lead to activation of glial cells have been proposed, including morphine binding to MORs, Toll-like receptor 4 (TLR4), ATP receptor P2X4, and chemokine receptors, among others [33, 45, 46]. Adding to the controversy, subsequent reports have found no change in tolerance in TLR4 knockout mice [48], and unequivocal evidence for MOR expression in glial cells is lacking [32]. Consequently, the contributions of neuronal versus glial cells and the molecular mechanisms initiating analgesic tolerance remain unresolved.

## 3. miRNAs and MORs

### 3.1. miRNAs and MOR Expression

With the deepening of research, people gradually realized that chronic morphine treatment would not alter the transcriptional ability of the MOR gene but may regulate the synthesis of MORs at the posttranscriptional level [18, 49]. The mechanism by which miRNAs regulate gene expression in animals is as follows: first, miRNAs are incorporated into the RNA-induced silencing complex (RISC), and then by complementary binding to 3′-UTR, the target mRNAs are recruited into the processing bodies, which do not contain the translational machinery. Next, mRNAs are sequestered or degraded by the decapping enzymes and exonucleases; consequently, the transcripts assembled on the ribosome for translation are reduced [18]. The MOR mRNA 3′-UTR is a relatively long noncoding region (in humans, it usually consist of more than 13,000 nucleotides) that is of great interest since this region may contain elements for the posttranscriptional regulation of receptor expression, such as altering the stability of the mRNA, influencing translational efficiency and controlling mRNA transport [50].

As early as 2008, studies have found that miR-23b can complementarily bind to the MOR mRNA 3′-UTR and reduce MOR expression at the posttranscriptional level. Further experiments showed that human neuroblastoma cells increased miR-23b expression in a dose- and time-dependent manner during chronic morphine exposure [14, 51]. Chronic morphine exposure increased miR-339-3p in mouse hippocampal neurons in vitro, which brought about destabilization and degradation of MOR mRNA by binding to a specific sequence of 3′-UTR that can partially be reversed by miR-339-3p inhibitor [15]. Another study has found that during morphine exposure in zebrafish embryos, activated MORs cause phosphorylation of cAMP-response element binding proteins (CREB; a protein that regulates the transcription of the miR-212/132 gene) via an extracellular signal-regulated kinase (ERK) or protein kinase A cascade signaling pathway, which then upregulated miR-212/132 and subsequently knocked down MOR mRNA expression through binding to 3′-UTR [16].

Most of the evidence has supported the opinion that morphine downregulates the expression of MORs, but there are also different accounts. It has been reported that miR-16 can bind to the 3′-UTR target site and weaken the translation of MOR mRNA, and morphine can upregulate MOR level by inhibiting the expression of miR-16, an effect reversed by the antagonist naloxone [17]. It needs to be emphasized that this finding is derived from the study of CEM ×174 cells (a kind of lymphocyte cell line), which is obviously different from the morphine tolerance of the nervous system we are going to explore. If the above cell-level studies are not sufficient to prove that miRNAs can modulate the expression of MORs in vivo, the following animal model studies can provide more precise evidence.

First, previous reports have found that the let-7 family of miRNAs has ubiquitous sequences capable of partial complementary binding to the MOR mRNA 3′-UTR [18, 52]. Chronic morphine-treated mice show marked upregulation of let-7 expression and let-7 production inhibitors effectively reduce morphine tolerance. Studies of zebrafish embryos treated with cocaine provide more evidence for let-7 involvement in MOR expression [53]. Similarly, a chronic subcutaneous implantation of morphine pellets in mice has resulted in miR-103 and miR-107 levels being significantly increased in the striatum with no change in the prefrontal cortex, and they both prevented MOR mRNA from assembling to the ribosome by binding to 3′-UTR [53]. miR-103 and miR-107 regulate overlapping targets and have identical sequences except for one nucleotide at the 3′-end, and both are transcribed from the introns of the pantothenate kinase family (PANK) genes. With regards to regional differential expression of miRNA103/107 after chronic morphine treatment, it is not clear whether the differential expression is related to the uneven distribution of MORs or other regulatory mechanisms.

At this point, we can conclude that chronic morphine treatment may upregulate the expression of certain miRNAs, which are partially complementary to and bind to the 3′-UTR of MORs mRNA to stop the translation of MORs rather than its degradation, resulting in decreasing MOR biosynthesis and aggravating morphine tolerance. Considering that a particular kind of miRNA can act on multiple target genes and a target gene may also be regulated by multiple miRNAs [3], the complexity of the posttranscriptional regulation of the MOR gene becomes clear. It is unclear, however, whether the abovementioned multiple miRNAs are specifically related to the development of morphine tolerance, but since they all bind to different targets on the mRNA 3′-UTR and regulate the expression of MORs, they may all play a role in morphine tolerance.

### 3.2. miRNAs and MOR Degradation

After knocking out the *β*-arrestin-2 (a G protein-coupled receptor regulatory protein) gene, the analgesic effect of morphine is significantly enhanced and prolonged, but morphine-induced analgesic tolerance, respiratory depression and acute constipation are greatly reduced [34, 35].

Recently, some researchers have conducted extensive research on the mechanism of *β*-arrestin-2 involvement in the development of morphine tolerance and have found that miR-365, which targets and inhibits the expression of *β*-arrestin-2, was significantly downregulated in the spinal cord of morphine-tolerant rats [9]. Note that lentivirus-mediated overexpression of miR-365 potently enhanced and prolonged morphine analgesia. A subsequent study also has reported similar results and suggested that ERK/CREB is an upstream regulatory pathway for morphine-induced downregulation of miR-365. miR-365 may also be involved in the activation of astrocytes and microglia because overexpressing miR-365 not only improved morphine analgesia but also reduced the release of glial cell activation-related cytokines [20].

Adding to the controversy, however, is that the expression of miR-365 in mice and rats hippocampal neurons is increased after chronic morphine or fentanyl exposure in vitro, and the activated MORs regulate the differential expression of multiple miRNAs via the ERK pathway [10]. It should be noted that this seemingly contradictory result is not particularly significant because morphine tolerance is a phenomenon that is apparent at the level of the entire body of the human or animal. Therefore, more experiments are needed to verify whether miR-365 is upregulated or downregulated in different tissues by chronic morphine exposure, as well as the relationship of any of these changes with morphine tolerance. A transcription factor that promotes the conversion of astrocytes to neurons is also regulated by miR-365 [54]. These findings once again remind us of the complexity of the regulation of miRNAs; that is, a miRNA may be involved in regulating the expression of multiple target genes.

## 4. miRNAs and Neuroadaptation

### 4.1. miRNAs and Synaptic Plasticity

The *N*-methyl-D-aspartate receptor (NMDAR) is mainly distributed in the synapses of the central nervous system, and brain-derived neurotrophic factor (BDNF) is mainly expressed in the nervous system [39, 55]. NMDAR and BDNF are well known as important molecules for LTP synaptic plasticity, and they play an important role in morphine tolerance [40, 55]. It has been reported that the BDNF scavenger tyrosine receptor kinase B-Fc (TrkB-Fc) relieved morphine tolerance in mice [21], and similarly, the NMDAR antagonists MK-801 (noncompetitive), LY235959 (competitive), and (+)-HA966 (glycine site-specific) significantly slowed the progression of morphine tolerance [56–58]. However, NMDAR antagonists are either ineffective or markedly neurotoxic in clinical practice [39].

BDNF is one of the well-known targets of miR-1, and the high expression of BDNF induced by the downregulation of miR-1 in the DRG has been considered a main cause of hyperalgesia and allodynia in rats with neuropathic pain [24]. Of note, miR-1 has been found to be downregulated in the prefrontal cortex of morphine-tolerant mice [5], which suggests that miR-1 may take part in morphine tolerance by directly targeting BDNF. In addition, there is evidence that neurons, astrocytes, and microglia may all secrete BDNF following stimulation by opioids, and consequently, BDNF regulates the expression of NMDAR [41, 55, 59].

A study of morphine tolerance in mice induced by subcutaneous injection of chronic morphine has found that as miR-219 levels gradually decreased, its target calcium/calmodulin-dependent protein kinase II gamma (CaMKII *γ*) and CaMKII *γ*-dependent BDNF expression gradually increased; this only occurred in the dorsal root ganglion (DRG) as there was no change in miR-219 and CaMKII *γ* in the corresponding segments of the spinal cord. Conversely, upregulation of miR-219 or downregulation of CaMKII *γ* and BDNF expression has been shown to be effective in reducing morphine tolerance in mice [21]. Another study of morphine tolerance in rats induced by intrathecal injection of chronic morphine came to similar conclusions; that is, miR-219 targeting CaMKII *γ* decreased NMDAR expression, which was regulated by the miR-219/CaMKII *γ* pathway [22]. Interestingly, two sets of studies have opposite results with respect to the localized expression of miR-219; miR-219 in the rat spinal cord (L4-L5) of the latter study gradually increased with analgesic tolerance, yet there was no change in the mouse spinal cord of the former study (L4–L6). Since the latter study did not investigate the expression of miR-219 in the rat DRG, we cannot, at this point, make conclusions on whether this contradictory result was related to differences in animal models and modes of drug administration, or whether there are other reasons.

At the same time when morphine induced analgesic tolerance by modulating BDNF expression as described above, another study found different regulatory pathways. By using the mouse morphine tolerance model mentioned above, miR-375 gradually decreased in the DRG as tolerance developed and the target Janus kinase 2 (JAK2) was upregulated, which then increased BDNF expression via the JAK2/signal transducer and activator of transcription 3 (STAT3) pathway. Adjusting any of the above nodes on these pathways has been shown to partially ameliorate morphine tolerance [23].

**Figure 1:**
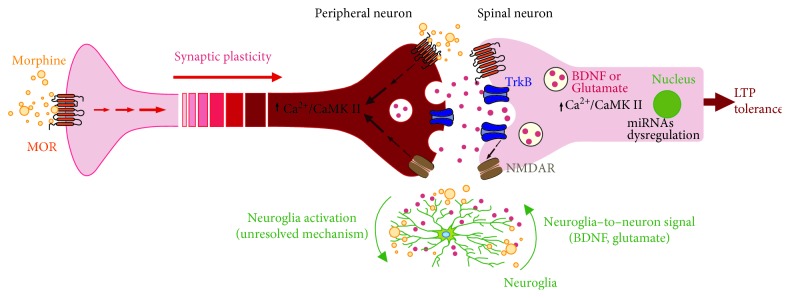
Schematic detailing the relationship among CaMKII-, BDNF-, and NMDAR-inducing LTP and morphine tolerance. Chronically activated MORs induce increases in Ca^2+^ levels and activation of the CaMKII signaling pathway at synapses, which subsequently triggers the release of BDNF and glutamate. On the one hand, BDNF directly induces LTP through downstream signaling, and, on the other hand, BDNF also upregulates the expression of NMDAR. An activated NMDAR signaling pathway not only directly induces LTP but also upregulates Ca^2+^ levels and activates the CaMKII signaling pathway. Finally, a mutual promotion circuit for CaMKII, BDNF, and NMDAR is formed and brings about morphine tolerance. Multiple miRNAs may be involved in regulating the circuit. MORs, *μ* opioid receptors; CaMKII, calcium/calmodulin-dependent protein kinase II; TrkB, tyrosine receptor kinase B; BDNF, brain-derived neurotrophic factor; miRNAs, microRNAs; LTP, long-term potentiation; NMDAR, *N*-methyl-D-aspartate receptor.

The above studies support the idea that morphine regulates the expression of CaMKII *γ*, BDNF, and NMDAR through miRNAs, leading to morphine tolerance. Collectively, the mutual relationship between CaMKII-, BDNF-, and NMDAR-inducing LTP is illustrated in Figure 1. Chronically activated MORs induce increases in Ca^2+^ level, and the CaMKII signaling pathway is activated at synapses [40, 41], which subsequently triggers the release of BDNF and glutamate. On one hand, BDNF directly induces LTP through downstream signaling, and on the other hand, BDNF also upregulates the expression of NMDAR. The activated NMDAR signaling pathway not only directly induces LTP but also upregulates Ca^2+^ level and activates the CaMKII signaling pathway [39, 41, 44]. Finally, a mutual promotion circuit with CaMKII, BDNF, and NMDAR is formed and brings about morphine tolerance. Multiple miRNAs may be involved in regulating the circuit.

### 4.2. miRNAs and Neuroplasticity

Changes in the number or structure of neurites caused by chronic morphine administration are thought to be involved in the development of morphine tolerance. miR-133b is thought to have the ability to promote neurite outgrowth and regulate neuroplasticity [25, 60]. Some researchers have suggested that miR-133b levels in rat hippocampal neurons in vitro was downregulated by the ERK pathway, which was activated via chronic morphine-activated MORs, which eventually caused abnormalities in synaptic signaling [25].

Neurogenic differentiation-1 (Neurod1) is an important transcription factor involved in the development and differentiation of neurons, and activated Neurod1 may contribute to dendritic spine stability, adult neurogenesis, learning and memory, and so forth [27]. Previous studies have found mechanistic differences in the effects of chronic morphine or fentanyl administration at MORs expressed by mouse hippocampal neurons in the regulation of Neurod1 activity. Both drugs reduced Neurod1 phosphorylation by inhibiting CaMKII*α* activity; however, fentanyl but not morphine, suppressed the expression of miR-190 by the ERK pathway and increased Neurod1 (negatively targeted by miR-190) protein levels. The final effect was that morphine reduced the overall activity of Neurod1, whereas fentanyl maintained it at the basal level [27]. Subsequent studies have found that morphine has a greater ability to induce analgesic tolerance in mice than fentanyl, which may be related to the mechanistic differences in the regulation of miR-190 levels. In addition, by reducing the stability of dendritic spines and inhibiting adult neurogenesis, the decrease in Neurod1 activity contributed to morphine tolerance in mice, which was partially mitigated by overexpressing Neurod1 [26].

Dicer, also known as endoribonuclease Dicer, can cleave pre-microRNA into microRNA. Serpin peptidase inhibitor clade-1 (Serpini1) is a serine protease activity regulator that intricately regulates dendritic spine density in creature and may have neuroprotective effects. Mechanistically, previous studies have proposed that Dicer is positively correlated with the development of morphine tolerance, but Serpini1 is negatively correlated with it [5]. In mouse prefrontal cortex, chronic morphine treatment has downregulated miR-27a, miR-146b, and miR-9 levels, yet upregulated miR-505 levels. Notably, on one hand, morphine tolerance decreased Serpini1 expression targeted positively by miR-27a, miR-146b, and miR-9 or negatively by miR-505; on the other hand, morphine tolerance increased Dicer expression targeted negatively by miR-27a, miR-146b, and miR-9 or positively by miR-505, respectively, at the posttranscriptional level [5].

## 5. Other Possible miRNAs

Other studies have suggested that miR-124 and miR-19b also target the expression of Neurod1 [27]. Notably, previous reports have found that the level of neuronal differentiation and the expression of glutamate transporter in human neural progenitor cells in vitro have been increased after exogenous miR-124 supplementation [61]. Synaptopodin, a key protein for synaptic transmission on the nociceptive pathway, is negatively targeted by miR-124. Moreover, others have shown that the reduction of miR-124 in mouse brain and spinal cord induced by neuropathic pain and bone cancer pain triggers the onset of microglial activation that results in persistent hyperalgesia, which can be prevented by intrathecal miR-124 administration [28, 62]. It is worth mentioning that miR-146a was downregulated in the spinal cord of rats with neuropathic pain, and tumor necrosis factor receptor-associated factor 6 (TRAF6) was upregulated as it targets and activates its downstream signaling pathways, leading to neuropathic pain [29].

A miRNA expression profile analysis of the mouse prefrontal cortex found that miR-19b and miR-146a levels are downregulated after morphine tolerance [5], but there are no detailed reports of miR-19b involvement in the regulation of nociceptive pathways. Given that morphine tolerance and hyperalgesia may share similar mechanisms [63, 64], such as the activation of microglia, we speculate that miR-124 and miR-146a may be involved in the development of morphine tolerance.

## 6. Conclusions

Opioid analgesia results from binding and signaling through MORs present along pain neural circuits; however, MOR-activated ERK/CREB signaling pathways lead to changes in the expression of multiple miRNAs, eventually resulting in the downregulation of MORs or neuromodulation. Although miRNAs provide a new starting point for revealing morphine tolerance mechanisms and preventing side effects, they also face many problems. First, morphine can cause a variety of miRNAs to up- or downregulate in the body, some of which have been shown to be involved in morphine tolerance, some of which may play a beneficial role in regulating analgesia, and some of which may mediate other adverse effects. Furthermore, there are obvious differences in the miRNA expression in distinct cells and the differential dysregulation of miRNAs caused by different opioids, which requires further study to identify the representative miRNAs that regulate morphine tolerance. Second, a miRNA can act on multiple target genes, and a target gene may also be regulated by multiple miRNAs. This diversity will create problems such as diagnostic specificity and treatment-related side effects when using miRNAs for clinical diagnosis and treatment.

Although only a small number of miRNA therapeutic clinical trials have been conducted and limited to Phase I studies for the treatment of cancer [65, 66], the concept of “therapeutic miRNA targeting” has attracted great interest. In summary, the molecular mechanisms of miRNAs participating in morphine tolerance are complex, and the use of miRNAs as targets for morphine tolerance may be a potential therapeutic approach but will face many challenges.

## Figures and Tables

**Table 1:** The possible miRNAs for morphine tolerance.

miRNAs	Model/site	Change after chronic morphine exposure	Target	Effect on tolerance after artificial regulation	References
miR-23b	Human neuroblastoma cells	Up	MOR	–	Wu et al. [14]
miR-339-3p	Mouse hippocampus cells	Up	MOR	Alleviation	Wu et al. [15]
miR-212/132	Zebrafish embryos	Up	MOR	–	Garcia-Concejo et al. [16]
miR-16	CEM ×174 cells	Down	MOR	–	Hou et al. [17]
Let-7a/7c/7g	Mouse, s.c., brain	Up	MOR	Alleviation	He et al. [18]
miR-103/107	Mouse, s.c., brain	Up	MOR	–	Lu et al. [19]
miR-365	Rat, i.t., spinal cord	Down	*β*-arrestin-2	Alleviation	Wang et al. [9]
					Wu et al. [20]
miR-219	Mouse, s.c., DRG	Down	CaMKII *γ*	Alleviation	Hu et al. [21]
miR-219	Rat, i.t., spinal cord	Down	CaMKII *γ*	Alleviation	Wang et al. [22]
miR-375	Mouse, s.c., DRG	Down	JAK2	Alleviation	Li et al. [23]
miR-1	Mouse, s.c., brain	Down	BDNF	–	Tapocik et al. [5]
					Neuman et al. [24]
miR-27a	Mouse, s.c., brain	Down	Serpini1, Dicer1	–	Tapocik et al. [5]
miR-146b	Mouse, s.c., brain	Down	Serpini1, Dicer1	–	Tapocik et al. [5]
miR-505	Mouse, s.c., brain	Up	Serpini1, Dicer1	–	Tapocik et al. [5]
miR-9	Mouse, s.c., brain	Down	Serpini1	–	Tapocik et al. [5]
miR-133b	Rat hippocampal neurons	Down	Pitx3	–	Sanchez-Simon et al. [25]
miR-190	Mouse, s.c., DRG	No	Neurod1	Alleviation	Li et al. [26]
miR-19b	Mouse, s.c., brain	Down	Neurod1	–	Tapocik et al. [5]
					Zheng et al. [27]
miR-124	Mouse, bone cancer pain, spinal cord	–	Synaptopodin, Neurod1	–	Elramah et al. [28]
					Zheng et al. [27]
miR-146a	miR-146a	Down	TRAF6	–	Tapocik et al. [5]
					Lu et al. [29]

s.c., subcutaneous; i.t., intrathecal; DRG, dorsal root ganglion; –, no research; MOR, *μ* opioid receptors; CaMKII *γ*, calcium/calmodulin-dependent protein kinase II gamma; JAK2, Janus kinase 2; BDNF, brain-derived neurotrophic factor; Serpini1, serpin peptidase inhibitor clade-1; Pitx3, paired-like homeodomain transcription factor 3; Neurod1, neurogenic differentiation-1; TRAF6, tumor necrosis factor receptor-associated factor 6.

## References

[1] Volkow N. D., McLellan A. T. (2016). Opioid abuse in chronic pain—misconceptions and mitigation strategies. *New England Journal of Medicine*.

[2] Bekhit M. H. (2010). Opioid-induced hyperalgesia and tolerance. *American Journal of Therapeutics*.

[3] Bartel D. P. (2004). MicroRNAs. *Cell*.

[4] Vasudevan S., Tong Y., Steitz J. A. (2007). Switching from repression to activation: microRNAs can up-regulate translation. *Science*.

[5] Tapocik J. D., Ceniccola K., Mayo C. L. (2016). MicroRNAs are involved in the development of morphine-induced analgesic tolerance and regulate functionally relevant changes in Serpini1. *Frontiers in Molecular Neuroscience*.

[6] Kosik K. S. (2006). The neuronal microRNA system. *Nature Reviews Neuroscience*.

[7] Im H.-I., Kenny P. J. (2012). MicroRNAs in neuronal function and dysfunction. *Trends in Neurosciences*.

[8] Toyama K., Kiyosawa N., Watanabe K., Ishizuka H. (2017). Identification of circulating miRNAs differentially regulated by opioid treatment. *International Journal of Molecular Sciences*.

[9] Wang J., Xu W., Zhong T. (2016). miR-365 targets *β*-arrestin 2 to reverse morphine tolerance in rats. *Scientific Reports*.

[10] Zheng H., Zeng Y., Zhang X., Chu J., Loh H. H., Law P.-Y. (2010). -Opioid receptor agonists differentially regulate the expression of mir-190 and NeuroD. *Molecular Pharmacology*.

[11] Christie M. J. (2008). Cellular neuroadaptations to chronic opioids: tolerance, withdrawal and addiction. *British Journal of Pharmacology*.

[12] Kieffer B. L., Evans C. J. (2002). Opioid tolerance-in search of the holy grail. *Cell*.

[13] Wei L.-N., Loh H. H. (2011). Transcriptional and epigenetic regulation of opioid receptor genes: present and future. *Annual Review of Pharmacology and Toxicology*.

[14] Wu Q., Zhang L., Law P.-Y., Wei L.-N., Loh H. H. (2009). Long-term morphine treatment decreases the association of -opioid receptor (MOR1) mRNA with polysomes through miRNA23b. *Molecular Pharmacology*.

[15] Wu Q., Hwang C. K., Zheng H. (2013). MicroRNA 339 down-regulates *μ*-opioid receptor at the post-transcriptional level in response to opioid treatment. *The FASEB Journal*.

[16] Garcia-Concejo A., Jimenez-Gonzalez A., Rodriguez R. E. (2016). Mu opioid receptor expression after morphine administration is regulated by mir-212/132 cluster. *PloS One*.

[17] Hou W., Li H., Jiang W., Zhang C., McNutt M. A., Li G. (2016). Simian immunodeficiency virus impacts microrna-16 mediated post-transcriptional regulation of mu opioid receptor in CEM ×174 cells. *Journal of Cellular Biochemistry*.

[18] He Y., Yang C., Kirkmire C. M., Wang Z. J. (2010). Regulation of opioid tolerance by let-7 family MicroRNA targeting the opioid receptor. *Journal of Neuroscience*.

[19] Lu Z., Xu J., Xu M., Pasternak G. W., Pan Y.-X. (2014). Morphine regulates expression of -opioid receptor MOR-1a, an intron-retention carboxyl terminal splice variant of the -opioid receptor (OPRM1) gene via miR-103/miR-107. *Molecular Pharmacology*.

[20] Wu X. P., She R. X., Yang Y. P., Xing Z. M., Chen H. W., Zhang Y. W. (2018). MicroRNA-365 alleviates morphine analgesic tolerance via the inactivation of the ERK/CREB signaling pathway by negatively targeting *β*-arrestin2. *Journal of Biomedical Science*.

[21] Hu X. M., Cao S. B., Zhang H. L. (2016). Downregulation of miR-219 enhances brain-derived neurotrophic factor production in mouse dorsal root ganglia to mediate morphine analgesic tolerance by upregulating CaMKII *γ*. *Molecular Pain*.

[22] Wang J., Xu W., Shao J. (2017). miR-219-5p targets CaMKII*γ* to attenuate morphine tolerance in rats. *Oncotarget*.

[23] Li H., Tao R., Wang J., Xia L. (2017). Upregulation of miR-375 level ameliorates morphine analgesic tolerance in mouse dorsal root ganglia by inhibiting the JAK2/STAT3 pathway. *Journal of Pain Research*.

[24] Neumann E., Hermanns H., Barthel F., Werdehausen R., Brandenburger T. (2015). Expression changes of microRNA-1 and its targets Connexin 43 and brain-derived neurotrophic factor in the peripheral nervous system of chronic neuropathic rats. *Molecular Pain*.

[25] Sanchez-Simon F. M., Zhang X. X., Loh H. H., Law P.-Y., Rodriguez R. E. (2010). Morphine regulates dopaminergic neuron differentiation via miR-133b. *Molecular Pharmacology*.

[26] Li W., He S., Zhou Y. (2014). Neurod1 modulates opioid antinociceptive tolerance via two distinct mechanisms. *Biological Psychiatry*.

[27] Zheng H., Law P. Y., Loh H. H. (2012). Non-coding RNAs regulating morphine function: with emphasis on the in vivo and in vitro functions of miR-190. *Frontiers in Genetics*.

[28] Elramah S., Lopez-Gonzalez M. J., Bastide M. (2017). Spinal miRNA-124 regulates synaptopodin and nociception in an animal model of bone cancer pain. *Scientific Reports*.

[29] Lu Y., Cao D.-L., Jiang B.-C., Yang T., Gao Y.-J. (2015). MicroRNA-146a-5p attenuates neuropathic pain via suppressing TRAF6 signaling in the spinal cord. *Brain, Behavior, and Immunity*.

[30] Matthes H. W. D., Maldonado R., Simonin F. (1996). Loss of morphine-induced analgesia, reward effect and withdrawal symptoms in mice lacking the *μ*-opioid-receptor gene. *Nature*.

[31] Arvidsson U., Riedl M., Chakrabarti S. (1995). Distribution and targeting of a mu-opioid receptor (MOR1) in brain and spinal cord. *Journal of Neuroscience*.

[32] Corder G., Tawfik V. L., Wang D. (2017). Loss of *μ* opioid receptor signaling in nociceptors, but not microglia, abrogates morphine tolerance without disrupting analgesia. *Nature Medicine*.

[33] Song P., Zhao Z.-Q. (2001). The involvement of glial cells in the development of morphine tolerance. *Neuroscience Research*.

[34] Raehal K. M., Walker J. K., Bohn L. M. (2005). Morphine side effects in *β*-arrestin 2 knockout mice. *Journal of Pharmacology and Experimental Therapeutics*.

[35] Bohn L. M., Gainetdinov R. R., Lin F.-T., Lefkowitz R. J., Caron M. G. (2000). *μ*-Opioid receptor desensitization by *β*-arrestin-2 determines morphine tolerance but not dependence. *Nature*.

[36] Nagi K., Pineyro G. (2011). Regulation of opioid receptor signalling: implications for the development of analgesic tolerance. *Molecular Brain*.

[37] Williams J. T., Ingram S. L., Henderson G. (2013). Regulation of -opioid receptors: desensitization, phosphorylation, internalization, and tolerance. *Pharmacological Reviews*.

[38] Kronschlager M. T., Drdla-Schutting R., Gassner M., Honsek S. D., Teuchmann H. L., Sandkuhler J. (2016). Gliogenic LTP spreads widely in nociceptive pathways. *Science*.

[39] Malenka R. C. (2003). The long-term potential of LTP. *Nature Reviews Neuroscience*.

[40] Drdla R., Gassner M., Gingl E., Sandkuhler J. (2009). Induction of synaptic long-term potentiation after opioid withdrawal. *Science*.

[41] Park H., Popescu A., Poo M.-M. (2014). Essential role of presynaptic NMDA receptors in activity-dependent BDNF secretion and corticostriatal LTP. *Neuron*.

[42] Sweatt J. D. (2016). Neural plasticity and behavior—sixty years of conceptual advances. *Journal of Neurochemistry*.

[43] Ma T., Cheng Y., Roltsch Hellard E. (2018). Bidirectional and long-lasting control of alcohol-seeking behavior by corticostriatal LTP and LTD. *Nature Neuroscience*.

[44] Zhou H.-Y., Chen S.-R., Chen H., Pan H.-L. (2010). Opioid-induced long-term potentiation in the spinal cord is a presynaptic event. *Journal of Neuroscience*.

[45] Watkins L. R., Hutchinson M. R., Rice K. C., Maier S. F. (2009). The “toll” of opioid-induced glial activation: improving the clinical efficacy of opioids by targeting glia. *Trends in Pharmacological Sciences*.

[46] Wen Y.-R., Tan P.-H., Cheng J.-K., Liu Y.-C., Ji R.-R. (2011). Microglia: a promising target for treating neuropathic and postoperative pain, and morphine tolerance. *Journal of the Formosan Medical Association*.

[47] Hua Z., Liu L., Shen J. (2016). Mesenchymal stem cells reversed morphine tolerance and opioid-induced hyperalgesia. *Scientific Reports*.

[48] Fukagawa H., Koyama T., Kakuyama M., Fukuda K. (2013). Microglial activation involved in morphine tolerance is not mediated by toll-like receptor 4. *Journal of Anesthesia*.

[49] Brodsky M., Elliott K., Hynansky A., Inturrisi C. E. (1995). CNS levels of mu opioid receptor (MOR-1) mRNA during chronic treatment with morphine or naltrexone. *Brain Research Bulletin*.

[50] Ide S., Han W., Kasai S., Hata H., Sora I., Ikeda K. (2005). Characterization of the 3′ untranslated region of the human mu-opioid receptor (MOR-1) mRNA. *Gene*.

[51] Wu Q., Law P.-Y., Wei L.-N., Loh H. H. (2008). Post-transcriptional regulation of mouse *μ* opioid receptor (MOR1) via its 3′ untranslated region: a role for microRNA23b. *The FASEB Journal*.

[52] He Y., Wang Z. J. (2012). Let-7 microRNAs and opioid tolerance. *Frontiers in Genetics*.

[53] Lopez-Bellido R., Barreto-Valer K., Sanchez-Simon F. M., Rodriguez R. E. (2012). Cocaine modulates the expression of opioid receptors and miR-let-7d in zebrafish embryos. *PloS One*.

[54] Mo J. L., Liu Q., Kou Z. W. (2018). MicroRNA-365 modulates astrocyte conversion into neuron in adult rat brain after stroke by targeting Pax6. *Glia*.

[55] Matsushita Y., Omotuyi I. O., Mukae T., Ueda H. (2013). Microglia activation precedes the anti-opioid BDNF and NMDA receptor mechanisms underlying morphine analgesic tolerance. *Current Pharmaceutical Design*.

[56] Trujillo K., Akil H. (1991). Inhibition of morphine tolerance and dependence by the NMDA receptor antagonist MK-801. *Science*.

[57] Adam F., Dufour E., Le Bars D. (2008). The glycine site-specific NMDA antagonist (+)-HA966 enhances the effect of morphine and reverses morphine tolerance via a spinal mechanism. *Neuropharmacology*.

[58] Mendez I. A., Trujillo K. A. (2008). NMDA receptor antagonists inhibit opiate antinociceptive tolerance and locomotor sensitization in rats. *Psychopharmacology*.

[59] Shen N., Mo L.-Q., Hu F., Chen P.-X., Guo R.-X., Feng J.-Q. (2014). A novel role of spinal astrocytic connexin 43: mediating morphine antinociceptive tolerance by activation of NMDA receptors and inhibition of glutamate transporter-1 in rats. *CNS Neuroscience & Therapeutics*.

[60] Xin H., Li Y., Buller B. (2012). Exosome-mediated transfer of miR-133b from multipotent mesenchymal stromal cells to neural cells contributes to neurite outgrowth. *Stem Cells*.

[61] Lee H. K., Finniss S., Cazacu S., Xiang C., Brodie C. (2014). Mesenchymal stem cells deliver exogenous miRNAs to neural cells and induce their differentiation and glutamate transporter expression. *Stem Cells and Development*.

[62] Willemen H. L., Huo X. J., Mao-Ying Q. L., Zijlstra J., Heijnen C. J., Kavelaars A. (2012). MicroRNA-124 as a novel treatment for persistent hyperalgesia. *Journal of Neuroinflammation*.

[63] Mayer D. J., Mao J., Holt J., Price D. D. (1999). Cellular mechanisms of neuropathic pain, morphine tolerance, and their interactions. *Proceedings of the National Academy of Sciences*.

[64] Mao J., Price D. D., Mayer D. J. (1995). Mechanisms of hyperalgesian and morphine tolerance: a current view of their possible interactions. *Pain*.

[65] Bouchie A. (2013). First microRNA mimic enters clinic. *Nature Biotechnology*.

[66] Janssen H. L. A., Reesink H. W., Lawitz E. J. (2013). Treatment of HCV infection by targeting microRNA. *New England Journal of Medicine*.

